# Autoimmune Regulator Gene Polymorphisms in Egyptian Systemic Lupus Erythematosus Patients: Preliminary Results

**DOI:** 10.1155/2021/5546639

**Published:** 2021-09-28

**Authors:** Doaa HS Attia, Dalia AH Dorgham, Ahmed A. El Maghraby, Marwa Alkaffas, Mahitab A. Abdel Kawy, Mai M. Sherif, Radwa M. Abdel Halim

**Affiliations:** ^1^Rheumatology and Rehabilitation Department, Cairo University, Egypt; ^2^Medical Biochemistry and Molecular Biology Department, Cairo University, Egypt; ^3^Clinical Pathology Department, Cairo University, Egypt; ^4^Chemical Pathology Department, Cairo University, Egypt

## Abstract

**Background:**

Systemic lupus erythematosus (SLE) is a systemic autoimmune disease. The autoimmune regulator (*AIRE*) is a master regulator of self-tolerance development. *AIRE* mutations lead to the development of autoimmune polyglandular syndrome type 1 while *AIRE* polymorphisms have been linked to organ-specific autoimmunity. The study is aimed at addressing the association between *AIRE* polymorphisms, rs2075876 (G > A) and rs760426 (A > G), and SLE susceptibility and expression in Egyptian patients.

**Methods:**

Ninety-nine patients were included. One hundred and ten, and 123 control subjects were genotyped for rs2075876 and rs760426, respectively. Lupus severity was assessed using the Lupus Severity of Disease Index and Lupus Severity Index (LSI). Systemic Lupus International Collaborating Clinics (SLICC)/American College of Rheumatology (ACR) damage index was considered. Genotyping was done using StepOne Real-Time PCR. *Results. AIRE* rs760426 GG was more frequent in the patients under the genotype level (14.1% vs. 4.9%, *p* = 0.032) and recessive model (14.1% vs. 4.9%, *p* = 0.017, OR = 3.2 (1.2-8.7)). Musculoskeletal involvement and nephritis were associated with *AIRE* rs2075876 under the dominant (97.9% vs. 80.8%, *p* = 0.009, OR = 11 (1.3-89.2)) and recessive models (100% vs. 69.3%, *p* = 0.032), respectively; and both were linked to *AIRE* rs2075876 at the allelic level: 98.3% vs. 85%, *p* = 0.005, OR = 10.1 (1.3-76.6) and 82.8% vs. 68.6, *p* = 0.041, OR = 2.2 (1-4.7), respectively. Patients with *AIRE* rs2075876 A alleles had a higher damage index ( 1 ± 1.3 vs. 0.6 ± 1.1, *p* = 0.045) while the LSI was greater in patients with *AIRE* rs2075876 (8.5 ± 0.5 vs. 7.8 ± 1.3, *p* = 0.002) and rs760426 (8.6 ± 11 vs. 7.8 ± 1.2, *p* = 0.031) under the recessive models. *Conclusion. AIRE* rs760426 could share in SLE susceptibility while *AIRE* rs2075876 could influence the disease expression and burden in Egyptian patients.

## 1. Introduction

Systemic lupus erythematosus (SLE) is a prototypic systemic autoimmune disease that is characterized by loss of self-tolerance with subsequent activation of the autoreactive T and B cells, production of autoantibodies, and eventual inflammation-induced tissue injury and organ dysfunction [1]. Being a common disease in the Arabs with a special predilection to the young productive population, it represents a great burden on the individual and national levels [2, 3].

Understanding the detailed pathogenesis of the disease helps the development of targeted therapies that are aimed at attacking the critical steps of disease pathogenesis, hence, aborting the disease process using more effective and less toxic medications [4]. It is hypothesized that SLE develops secondary to the effect of environmental factors on a genetically predisposed person. Several human leucocytic antigen (HLA) and non-HLA genes have been suggested as risk factors for SLE [5].

The autoimmune regulator (*AIRE*) is a transcriptional factor that controls the expression of peripheral autoantigens in the thymus enhancing the negative selection of the autoreactive T lymphocytes during the process of thymic education [6, 7]. Mutations of the *AIRE* gene lead to the development of autoimmune polyglandular syndrome type 1 (APS1) or autoimmune polyendocrinopathy candidiasis-ectodermal dystrophy syndrome: a systemic autoimmune disease characterized by the development of Addison's disease, hypoparathyroidism, and chronic mucocutaneous candidiasis [8, 9].

Additional evidence of the utmost importance of *AIRE* in self-tolerance development is the link between *AIRE* gene polymorphisms and several autoimmune disorders [10] including vitiligo [11], type 1 diabetes [12], Hashimoto's thyroiditis [13], Graves' disease [14], autoimmune hepatitis [15], myasthenia gravis [16], and alopecia areata [17]. Moreover, *AIRE*-deficient mice could express a wide spectrum of autoimmune phenomena depending on the genetic background of the strain [18].

Being a prototypic systemic autoimmune disease, our study is aimed at addressing the frequency of *AIRE* single-nucleotide polymorphisms (SNPs) rs2075876 (G>A) and rs760426 (A>G) in SLE patients compared with the controls, and their relation to the phenotypic disease expression in an Egyptian cohort. *AIRE* rs2075876 and rs760426 were particularly selected for the study as systematic reviews and meta-analyses showed their significant association with RA [19–24]: another systemic immune complex-mediated autoimmune disease showing common genetic risk factors with SLE [25]. We hypothesized that *AIRE* SNPs could be implicated in systemic autoimmunity [18] as *AIRE* dysfunction could influence organs of different natures and functions, i.e., the skin, endocrine glands, and liver [10–17]. To the best of knowledge, few studies involving *AIRE* polymorphisms in lupus patients were found to date in the literature [26, 27].

## 2. Materials and Methods

Patients enrolled in this case-control study were recruited from the Rheumatology and Rehabilitation Department, Kasr AL-Ainy Hospital, Cairo University, Egypt. The control subjects had no family history of autoimmune diseases. Patients were classified/reclassified according to the 2012 Systemic Lupus International Collaborating Clinics (SLICC) classification criteria for SLE [28].

Demographic, clinical, and investigational data were collected from patients' charts using a standardized form. Disease onset was defined as the time point of the development of the first disease manifestation. Disease duration was defined as the duration between the disease onset and the time of enrolment. Lupus severity was assessed using Lupus Severity of Disease Index (Lupus SDI) [29] and Lupus Severity Index (LSI) [30]. SLICC/ACR damage index [31] was considered as well.

All data were studied under the genetic models, and the genotype and allelic distributions. The genetic models were determined based on the studied SNP.

The study conformed to the provisions of the Declaration of Helsinki. The local ethics committee approval was obtained (N9-2018). All participants gave informed consent.

No study had addressed *AIRE* SNPs in SLE patients when this study was designed. Hence, sample size calculation was not possible and a pilot study involving ten patients and ten controls was commenced. The frequency of *AIRE* rs760426 under the dominant model was 30% in patients and 0% in controls. Using these results with an alpha error of 0.05 and a power of 95%, a minimum sample size of 33 patients and 33 controls was required.

### 2.1. DNA Extraction and Genotyping

According to the manufacturer's recommendations, genomic DNA was isolated from the whole blood using a QIAamp DNA blood mini kit (Qiagen, Germany) and stored at -20°C.

Genotyping of the rs2075876 and rs760426 of the *AIRE* gene was done across all participants' sample sets using the TaqMan Allelic Discrimination Assay Kit (probe ID C__15863141_20 and C__5886344_10, respectively, Applied Biosystems, Foster City, CA, USA), and these SNPs were analyzed on StepOne Real-Time Polymerase Chain Reaction System (Applied biosystems CA94404, Foster City, USA).

### 2.2. Statistical Methods

The Hardy–Weinberg equilibrium (HWE) online calculator was used for the calculation of the allelic frequencies [32]. Haplotype frequency was calculated using the SNPStats online calculator [33]. The data were tabulated and statistically analyzed. Results were described in terms of mean ± standard deviation (± SD) or median and interquartile range (IQR) for quantitative data, and numbers and percentages for qualitative data. Statistical differences between groups were tested using the Chi-square tests for qualitative variables; Bonferroni adjustment was applied for multiple comparisons setting a new *p* value of 0.017 for 3 comparisons (between AA and AG, AA and GG, and AG and GG for each of rs2075876 and rs760426 SNPs). For the quantitative data, the unpaired sample *t*-test was used for the comparison between two groups. One-way analysis of variance (ANOVA) was used for comparing numerical data between multiple groups while the Kruskal-Wallis test was used instead when the homogeneity of variance assumption was violated. Genotype frequencies were compared between the disease and control groups using logistic regression; odds ratio (OR) with 95% confidence intervals was calculated. A two-tailed probability value (*p* value) less than 0.05 was considered statistically significant unless specified. Statistical analyses were performed using the Statistical Package for the Social Science (SPSS) 20.0 statistical package.

## 3. Results

The study included 99 SLE patients, 92 female and 7 male patients. Their mean age of disease onset was 23.32 ± 8.62 years, the mean age at recruitment was 30.91 ± 8.55 years, and the median disease duration was 7 years (IQR: 3-13 years). One hundred and ten, and 123 control subjects were genotyped for rs2075876 and rs760426, respectively, with a total of 109 (88.6%) females and 14 (11.4%) males and a mean age of 31.79 ± 5.34 years. Both the patient and control groups were matched for age, sex, and ethnicity.

**Figure 1:**
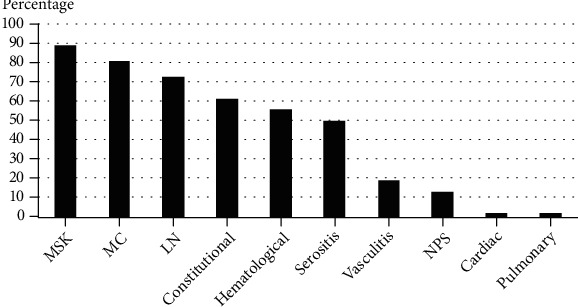
The frequency of the different disease phenotypes in the lupus cohort. MSK: musculoskeletal; MC: mucocutaneous; LN: lupus nephritis; NPS: neuropsychiatric.

**Figure 2:**
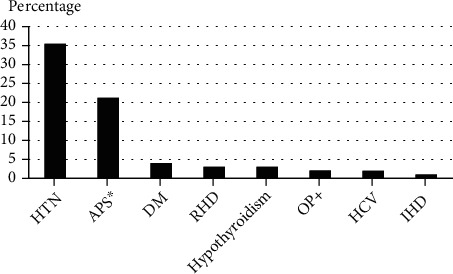
The frequency of comorbidities among the lupus cohort. HTN: systemic hypertension; APS: antiphospholipid syndrome; DM: diabetes mellitus; RHD: rheumatic heart disease; OP: osteoporosis; HCV: hepatitis C virus; IHD: ischemic heart disease. ^∗^The Sydney classification criteria were used [41]. ^†^Diagnosis of osteoporosis was based on a patient's low bone mineral density in comparison to that of a healthy adult of matched age and sex plus the presence of an osteoporotic fracture(s) [42].

The frequencies of the clinical disease manifestations and the associated comorbidities among the study cohort are illustrated in Figures 1 and 2, respectively. The most common clinical features were musculoskeletal (88.9%) and mucocutaneous (80.8%). Regarding major organ involvement, lupus nephritis and neuropsychiatric manifestations developed in 72.7% and 13.1% of patients, respectively. The most common associated comorbidities in the study cohort were systemic hypertension (35.4%) and antiphospholipid syndrome (21.2%); other comorbidities represented minorities.

The genotype, genetic models, and allele frequencies of *AIRE* rs2075876 in the patients and control groups are shown in Table 1. The A allele of *AIRE* rs2075876 was more frequently found in the patients group compared with the control group (29.3% vs. 20.9%, *p* = 0.048, OR = 1.6 (95% CI: 1-2.4)).

The genotype and allele frequencies of *AIRE* rs760426 in the patients and control groups are shown in Table 2. Under the genotype distribution, the homozygous SNP was more common in the patients group compared with the control group (14.1% vs. 4.9%, *p* = 0.032), and this significant difference was persistent after Bonferroni correction. Logistic regression using the AA genotype as the reference group revealed that the GG genotype was statistically more common in the patients group compared with the control group (*p* = 0.037, OR: 2.934 (95% CI: 1.069-8.053)). Moreover, *AIRE* rs760426 was more prevalent in the patients group under the recessive model (*p* = 0.017, OR: 3.2 (95% CI: 1.2-8.7)).

The distribution of the genotypes in the control subjects was in accordance with Hardy–Weinberg equilibrium for both polymorphisms: the Chi-square values are <3.2 for both SNPs with a *p* value > 0.05.

Regarding frequencies of *AIRE* gene (rs2075876 and rs760426) haplotypes in the study group and the association with SLE, the A allele (in rs2075876 SNP) and the G allele (in rs760426 SNP) were coexistent in 54% of SLE patients in contrast to 46% of controls with a significant difference (*p* value = 0.014; OR = 10.95 (1.65-72.62)). Linkage disequilibrium analysis revealed that there is no linkage between the two studied *AIRE* gene SNPs (rs2075876 and rs760426) (Supplementary table 1).

The association between *AIRE* rs2075876 and rs760426 and the different disease parameters in the patients group are shown in Tables 3 and 4, respectively. The wild genotype of *AIRE* rs2075876 was more frequently observed in patients without musculoskeletal involvement, and this significance was persistent after Bonferroni correction (19.2%, 2.8%, and 0% for the GG, AG, and AA genotypes, respectively). After logistic regression, 80.7% of patients with the GG genotype had musculoskeletal involvement compared with 97.2% of patients with the AG genotype (*p* = 0.022, OR: 0.12 (0.015-0.984)). Under the dominant model, *AIRE* rs2075876 AA+AG was more frequent in patients with musculoskeletal involvement (97.9% for AA+AG versus 80.8% for GG) with a *p* value of 0.009, OR = 11 (95% CI: 1.3-89.2).

In addition, *AIRE* rs2075876 AA was more common in patients with lupus nephritis (LN) under the recessive model (100% for AA versus 69.3% for AG+GG) with a *p* value of 0.032. Both, musculoskeletal involvement and LN, were more common in patients with the A alleles compared with patients with the G alleles: 98.3% versus 85% for musculoskeletal affection and 82.8% versus 68.6% for nephritis with *p* values of 0.005 and 0.041, and OR (95% CI) of 10.1 (1.3-76.6) and 2.2 (1-4.7), respectively.

The SLICC/ACR damage index was significantly higher in patients with the A alleles of the *AIRE* rs2075876 (1 ± 1.3) compared with patients with the G alleles (0.6 ± 1.1), with a *p* value of 0.045; notably, patients within the A and G groups had comparable disease durations (8.2 ± 9.3 years versus 9.5 ± 8.1 years, respectively, *p* = 0.344). Moreover, the disease severity, as assessed by the LSI, was significantly higher in patients with *AIRE* rs2075876 and rs760426 variant allele homozygosity under the recessive models (8.5 ± 0.5 vs. 7.8 ± 1.3, *p* = 0.002 and 8.6 ± 11 vs. 7.8 ± 1.2, *p* = 0.031, respectively).

As a family history of SLE was reported in only 4 cases belonging to 4 different families, a statistical analysis of this finding was not feasible.

## 4. Discussion

Although *AIRE* polymorphisms are associated with the development of organ-specific autoimmunity [11–17], the study of this topic in systemic autoimmune diseases seems to be interesting. Several studies addressed *AIRE* gene polymorphisms in RA patients [19–22], and one study tackled it in patients with progressive systemic sclerosis (PSS) [34]. To the best of our knowledge, only two studies recently addressed *AIRE* SNPs in SLE patients [26, 27].

Our study is aimed at addressing the association between *AIRE* SNPs, rs2075876 (G>A) and rs760426 (A>G), and SLE: a prototypic systemic autoimmune disease.

Regarding the association with the disease susceptibility, *AIRE* rs760426 was particularly associated with disease occurrence. Moreover, the homozygous SNP of *AIRE* rs760426 was more common in the patients group compared with the control group (14.1% vs. 4.9%, *p* = 0.032) under the genotype distribution and this significant difference was persistent after Bonferroni correction. Logistic regression using the AA genotype as the reference group revealed that the GG genotype was statistically more common in the patients group compared with the control group (*p* = 0.037, OR: 2.934 (95% CI: 1.069-8.053)). Moreover, *AIRE* rs760426 was more prevalent in the patients group under the recessive model (*p* = 0.017, OR: 3.2 (95% CI: 1.2-8.7)).

Concerning the relation to organ system involvement, *AIRE* rs2075876 was the main SNP of the two related to the disease expression. The wild genotype of *AIRE* rs2075876 was more frequently observed in patients without musculoskeletal involvement, and this significance was persistent after Bonferroni correction (19.2%, 2.8%, and 0% for the GG, AG, and AA genotypes, respectively). After logistic regression, 80.7% of patients with the GG genotype had musculoskeletal involvement compared with 97.2% of patients with the AG genotype (*p* = 0.022, OR: 0.12 (0.015-0.984)). Under the dominant model, *AIRE* rs2075876 AA+AG was more frequent in patients with musculoskeletal involvement (97.9% for AA+AG versus 80.8% for GG) with a *p* value of 0.009, OR = 11 (95% CI: 1.3-89.2).

In addition, *AIRE* rs2075876 AA was more common in patients with lupus nephritis (LN) under the recessive model (100% for AA versus 69.3% for AG+GG) with a *p* value of 0.032. Both, musculoskeletal involvement and LN, were more common in patients with the A alleles compared with those with the G alleles: 98.3% versus 85% for musculoskeletal affection and 82.8% versus 68.6% for nephritis, with *p* values of 0.005 and 0.041, respectively. These observations could suggest the involvement of *AIRE* rs2075876 in disease phenotypic expression.

In relation to the burden of the disease, the SLICC/ACR damage index was significantly higher in patients with the A alleles of the *AIRE* rs2075876 (1 ± 1.3) compared with the G alleles (0.6 ± 1.1), with a *p* value of 0.045; notably, the A and G alleles patients groups had comparable disease durations (8.2 ± 9.3 years versus 9.5 ± 8.1 years, *p* = 0.344). Moreover, the disease severity, as assessed by the LSI, was significantly higher in patients with *AIRE* rs2075876 and rs760426 variant allele homozygosity under the recessive models.

In a study of *AIRE* rs2075876 G>A and *AIRE* rs878081 C>T in Mexican SLE patients, there was no association between *AIRE* rs2075876 and SLE disagreeing with our findings. On the other side, *AIRE* rs878081, a SNP that was not tackled in this study, was identified as a susceptibility variant for SLE. In discordance with the study results, neither *AIRE* rs2075876 nor *AIRE* rs878081 was associated with the occurrence of LN [26]. The controversy between the results of this Mexican study and ours could be explained by differences concerning genetics and environmental exposures in addition to the polygenic nature of the disease, i.e., the predisposition to and severity of SLE could be influenced by different genes in different populations.

Contrary to the Mexican study, a recent study involving Egyptian patients reported that *AIRE* rs2075876 variant seems protective against SLE development under the allelic and dominant models while patients with the *AIRE* rs2075876 AA genotype had statistically significant lower levels of C3 [27].

Polymorphisms of the *AIRE* gene were studied as risk variants for RA in several studies. In a genome-wide association (GWA) study involving Japanese patients with RA, *AIRE* rs2075876 and rs760426 showed significant associations with the disease [29]. In another study of Chinese RA patients, significant associations with RA were observed for *AIRE* rs2075876 under the genotypic and allelic distributions and under the recessive model. While there was a tendency of a higher frequency of the G alleles of *AIRE* rs760426 in the patients compared with the controls, there was a significant association between *AIRE* rs760426 and the disease under the recessive model [20].

In a third study of 9 SNPs of the *AIRE* gene in Han Chinese RA population including *AIRE* rs2075876, rs2075877, rs933150, rs1003854, rs2256817, rs3746964, rs878081, rs760426, and rs1078480, the study revealed an increased prevalence of the minor allele A of *AIRE* rs2075876 in the patients compared with the control group. Moreover, there was a significant association between *AIRE* rs2075876 and RA under the codominant and dominant models. On the other hand, *AIRE* rs933150 and rs760426 showed a borderline association with RA [21].

Another study of five SNPs of the *AIRE* gene was carried out in European RA patients. While there was no significant association between *AIRE* rs2776377, rs2075876, rs1055311, and rs1800520 SNPs and RA, the C variant of rs878081 was identified as a risk variant. Moreover, RA patients showed a higher frequency of *AIRE* rs878081 variant allele homozygosity. An interesting finding of this study is the association between *AIRE* rs878081, a synonymous allele, and a lower expression rate of the *AIRE* gene, as detected by the in silico analysis [24]. The authors explained this finding by the lower affinity of nuclear factor kappa B, an important transcriptional factor for the *AIRE* gene expression, to this variant allele [35, 36].

In a systematic review and meta-analysis study including case-control studies of the *AIRE* gene SNPs rs2075876 (G>A) and rs760426 (A>G) in RA patients, both SNPs were identified as risk variants for RA under all the genetic models. As most of the involved studies were derived from the Far Eastern populations, the authors could not extrapolate these results to the Caucasians [24]. Another meta-analysis of the *AIRE* rs2075876 revealed that this SNP increased the risk of RA under all genetic models. In the subgroup analysis of this study, *AIRE* rs2075876 increased RA susceptibility among Asians but not among Caucasians [25].

Concerning PSS, another *AIRE* SNP, G11107A, was linked to the occurrence of autoimmune thyroiditis in PSS patients [34].

The discrepancies between Asians and Caucasians in the aforementioned studies were suggested to be attributed to the racial difference of the minor allele frequencies [37], the ethnicity-specific effect of the SNPs, the different environmental exposures, the multifactorial etiology of the disease, the clinical disease heterogeneity, and the small sample size of some studies [25].

Despite the fact that rs2075876 (G>A) and rs760426 (A>G) are located in the intronic region, polymorphisms of *AIRE* gene noncoding regions have been reported to impair the thymic negative selection, hence, increasing the susceptibility to autoimmune diseases [38]. These observations could be explained by the intron-mediated decrement of the gene expression [39, 40].

Although SLE is a polygenic disease that is influenced by several environmental factors [5], this study seems to add to the genetic background of the disease. The study limitations include the lack of evaluation of associations between the studied polymorphisms and the level of expression of *AIRE*. Being a study with some marginally significant *p* values and some wide confidence intervals, further studies, including *AIRE* gene sequencing and expression, on different ethnicities and including a larger sample size are recommended to clarify the association between *AIRE* polymorphisms, and disease susceptibility, phenotypic expression, and severity. Studying *AIRE* rs2075876 (G>A) and rs760426 (A>G) could pave the way to genome-wide association studies as the next step to discover other *AIRE* gene key polymorphisms associated with SLE.

## 5. Conclusion

The susceptibility to SLE in Egyptian patients could be linked to *AIRE* rs760426 (A>G) while the phenotypic expression and burden of the disease could be related to *AIRE* rs2075876 (G>A).

## Figures and Tables

**Table 1:** Genotype and allele frequency of *AIRE* rs2075876 in the lupus patients and control groups.

			Patients (*n* = 99)	Control (*n* = 110)	*p* value	OR (95% CI)
Genotype		AA, *n* (%)	11 (11.1)	8 (7.3)	0.159	
		AG, *n* (%)	36 (36.4)	30 (27.3)		
		GG, *n* (%)	52 (52.5)	72 (65.5)		
Models	Dominant	AA+AG, *n* (%)	47 (47.5)	38 (34.5)	0.057	1.7 (1-3)
		GG, *n* (%)	52 (52.5)	72 (65.5)		
	Recessive	AA, *n* (%)	11 (11.1)	8 (7.3)	0.335	1.6 (0.6-4.1)
		GG+AG, *n* (%)	88 (88.9)	102 (92.7)		
			Patients (2*n* = 198)	Control (2*n* = 220)		
Allele		A, *n* (%)	58 (29.3)	46 (20.9)		
					0.048	1.6 (1-2.4)
		G, *n* (%)	140 (70.7)	174 (79.1)		

The comparisons were done using the Chi-square tests. *n*: number; OR: odds ratio; CI: confidence interval.

**Table 2:** Genotype and allele frequency of *AIRE* rs760426 in the lupus patients and control groups.

			Patients (*n* = 99)	Control (*n* = 123)	*p* value	OR (95% CI)
Genotypes		GG, *n* (%)	14 (14.1)	6 (4.9)	0.032^∗^	
		AG, *n* (%)	19 (19.2)	34 (27.6)		
		AA, *n* (%)	66 (66.7)	83 (67.5)		
Models	Dominant	GG+AG, *n* (%)	33 (33.3)	40 (32.5)	0.898	1 (0.6-1.8)
		AA, *n* (%)	66 (66.7)	83 (67.5)		
	Recessive	GG, *n* (%)	14 (14.1)	6 (4.9)	0.017	3.2 (1.2-8.7)
		AA+AG, *n* (%)	85 (85.9)	117 (95.1)		
			Patients (2*n* = 198)	Control (2*n* = 246)		
Alleles		G, *n* (%)	47 (23.7)	46 (18.7)	0.195	1.4 (0.9-2.1)
		A, *n* (%)	151 (76.3)	200 (81.3)		

The comparisons were done using the Chi-square tests. ^∗^After Bonferroni correction and logistic regression, the GG genotype was significantly more frequent in the patients group than the control group. *n*: number, OR: odds ratio, CI: confidence interval.

**Table 3:** The association between *AIRE* rs2075876 and the clinical parameters in the lupus patients.

	Genotypes				*Models*								Alleles			
					*Dominant*				Recessive							
	AA (*n* = 11)	AG (*n* = 36)	GG (*n* = 52)	*p* value	AA+AG (*n* = 47)	GG (*n* = 52)	*p* value	OR (95% CI)	AA (*n* = 11)	GG+AG (*n* = 88)	*p* value	OR (95% CI)	A (2*n* = 58)	G (2*n* = 140)	*p* valve	OR (95% CI)
Constitutional, *n* (%)	8 (72.7)	25 (69.4)	28 (53.8)	0.242	33 (70.2)	28 (53.8)	0.094	2.0 (0.9-4.6)	8 (72.7)	53 (60.2)	0.523	1.8 (0.4-7.1)	41 (70.7)	81 (57.9)	0.091	1.8 (1-3.4)
MC, *n* (%)	8 (72.7)	32 (88.9)	40 (76.9)	0.289	40 (85.1)	40 (76.9)	0.302	1.7 (0.6-4.8)	8 (72.7)	72 (81.8)	0.437	0.6 (0.1-2.5)	48 (82.8)	112 (80)	0.654	1.2 (0.5-2.7)
MSK, *n* (%)	11 (100)	35 (97.2)	42 (80.8)	0.025^a^	46 (97.9)	42 (80.8)	0.009	11 (1.3-89.2)	11 (100)	77 (87.5)	0.606		57 (98.3)	119 (85)	0.005	10.1 (1.3-76.6)
Serositis, *n* (%)	3 (27.3)	20 (55.6)	26 (50)	0.258	23 (48.9)	26 (50)	0.916	1 (0.4-2.1)	3 (27.3)	46 (52.3)	0.200	0.3 (0.1-1.4)	26 (44.8)	72 (51.4)	0.398	0.8 (0.4-1.4)
Nephritis, *n* (%)	11 (100)	26 (72.2)	35 (67.3)	0.086	37 (78.7)	35 (67.3)	0.203	1.8 (0.7-4.5)	11 (100)	61 (69.3)	0.032		48 (82.8)	96 (68.6)	0.041	2.2 (1-4.7)
Hematological, *n* (%)	6 (54.5)	17 (47.2)	32 (61.5)	0.413	23 (48.9)	32 (61.5)	0.208	0.6 (0.3-1.3)	6 (54.5)	49 (55.7)	0.943	1 (0.3-3.4)	29 (50)	81 (57.9)	0.311	0.7 (0.4-1.3)
NPS, *n* (%)	2 (18.2)	5 (13.9)	6 (11.5)	0.827	7 (14.9)	6 (11.5)	0.622	1.3 (0.4-4.3)	2 (18.2)	11 (12.5)	0.635	1.6 (0.3-8.2)	9 (15.5)	17 (12.1)	0.522	1.3 (0.6-3.2)
Cardiac, *n* (%)	1 (9.1)	1 (2.8)	0 (0)	0.138	2 (4.3)	0 (0)	0.223		1 (9.1)	1 (1.1)	0.211	8.7 (0.5-150.1)	3 (5.2)	1 (0.7)	0.076	7.6 (0.8-74.5)
Pulmonary, *n* (%)	1 (9.1)	0 (0)	1 (1.9)	0.172	1 (2.1)	1 (1.9)	>0.999	1.1 (0.07-18.2)	1 (9.1)	1 (1.1)	0.211	8.7 (0.5-150.1)	2 (3.4)	2 (1.4)	0.582	2.5 (0.3-17.9)
Vasculitis, *n* (%)	2 (18.2)	6 (16.7)	11 (21.2)	0.867	8 (17)	11 (21.2)	0.602	0.8 (0.3-2.1)	2 (18.2)	17 (19.3)	>0.999	1 (0.2-4.7)	10 (17.2)	28 (20)	0.654	0.8 (0.4-1.9)
SDI (mean ± SD)	5.6 ± 1.8	5.3 ± 2.2	4.92 ± 1.9	0.501	5.4 ± 2.1	4.9 ± 1.9	0.255		5.6 ± 1.8	5.1 ± 2	0.480		5.4 ± 2	5 ± 2	0.217	
LSI (mean ± SD)	8.5 ± 0.5	7.8 ± 1.2	7.8 ± 1.3	0.179^b^	8 ± 1.1	7.8 ± 1.3	0.573		8.5 ± 0.5	7.8 ± 1.3	0.002		8.1 ± 1	7.8 ± 1.3	0.155	
SLICC damage index (mean ± SD)	1.2 ± 1.5	0.9 ± 1.3	0.5 ± 1	0.170	1 ± 1.3	0.5 ± 1	0.088		1.2 ± 1.5	0.7 ± 1.1	0.169		1 ± 1.3	0.6 ± 1.1	0.045	

Comparisons were done using the Chi-square tests for the categorical data while the unpaired sample *t*-test and the one-way ANOVA test were used to compare the quantitative data between two and multiple groups, respectively, unless specified. ^a^The significant difference was persistent after Bonferroni correction and logistic regression. ^b^Kruskal-Wallis test was used. *n*: number. MC: mucocutaneous; MSK: musculoskeletal; NPS: neuropsychiatric; SDI: Lupus Severity of Disease Index; LSI: Lupus Severity Index; SLICC: Systemic Lupus International Collaborating Clinics; OR: odds ratio; CI: confidence interval.

**Table 4:** The association between AIRE rs760426 and the clinical parameters in the lupus patients.

	Genotypes				Models								Alleles			
					Dominant				Recessive							
	GG (*n* = 14)	AG (*n* = 19)	AA (*n* = 66)	*p* value	GG+AG (*n* = 33)	AA (*n* = 66)	*p* value	OR (95% CI)	GG (*n* = 14)	AA+AG (*n* = 85)	*p* value	OR (95% CI)	G (2*n* = 47)	A (2*n* = 151)	*p* value	OR (95% CI)
Constitutional, *n* (%)	12 (85.7)	12 (63.2)	37 (56.1)	0.115	24 (72.7)	37 (56.1)	0.108	2.1 (0.8-5.2)	12 (85.7)	49 (57.6)	0.073	4.4 (0.9-20.9)	34 (72.3)	88 (58.3)	0.083	1.9 (1-3.8)
MC, *n* (%)	11 (78.6)	18 (94.7)	51 (77.3)	0.228	29 (87.9)	51 (77.3)	0.282	2.1 (0.6-7)	11 (78.6)	69 (81.2)	0.729	0.9 (0.2-3.4)	41 (87.2)	119 (78.8)	0.200	1.8 (0.7-4.7)
MSK, *n* (%)	12 (85.7)	19 (100)	57 (86.4)	0.229	31 (93.9)	57 (86.4)	0.327	2.4 (0.5-12)	12 (85.7)	76 (89.4)	0.652	0.7 (0.1-3.7)	43 (91.5)	133 (88.1)	0.606	1.5 (0.5-4.5)
Serositis, *n* (%)	9 (64.3)	10 (52.6)	30 (45.5)	0.421	19 (57.6)	30 (45.5)	0.255	1.6 (0.7-3.8)	9 (64.3)	40 (47.1)	0.232	2 (0.6-6.5)	27 (57.4)	71 (47.0)	0.212	1.5 (0.8-3)
Nephritis, *n* (%)	11 (78.6)	14 (73.7)	47 (71.2)	0.849	25 (57.8)	47 (71.2)	0.632	1.3 (0.5-3.3)	11 (78.6)	61 (71.8)	0.752	1.4 (0.4-5.6)	35 (74.5)	109 (72.2)	0.759	1.1 (0.5-2.4)
Hematological, *n* (%)	7 (50)	10 (52.6)	38 (57.6)	0.840	17 (51.5)	38 (57.6)	0.567	0.8 (0.3-1.8)	7 (50)	48 (56.5)	0.652	0.8 (0.2-2.4)	23 (48.9)	87 (57.6)	0.296	0.7 (0.4-1.4)
NPS, *n* (%)	3 (21.4)	2 (10.5)	8 (12.1)	0.601	5 (15.2)	8 (12.1)	0.674	1.3 (0.4-4.3)	3 (21.4)	10 (11.8)	0.388	2 (0.5-8.6)	9 (19.1)	17 (11.3)	0.162	1.9 (0.8-4.5)
Cardiac, *n* (%)	0 (0)	1 (5.3)	1 (1.5)	0.501	1 (3)	1 (1.5)	>0.999	2 (0.1-33.5)	0 (0)	2 (2.4)	>0.999		0 (0)	4 (2.6)	0.574	
Pulmonary, *n* (%)	0 (0)	1 (5.3)	1 (1.5)	0.501	1 (3)	1 (1.5)	>0.999	2 (0.1-33.5)	0 (0)	2 (2.4)	>0.999		2 (4.3)	2 (1.3)	0.239	3.3 (0.5-24.2)
Vasculitis, *n* (%)	1 (7.1)	4 (21.1)	14 (21.2)	0.466	5 (15.2)	14 (21.2)	0.470	0.7 (0.2-2)	1 (7.1)	18 (21.2)	0.294	0.3 (0-2.3)	5 (10.6)	33 (21.9)	0.088	0.4 (0.2-1.2)
SDI (mean ± SD)	5.5 ± 1.9	5.2 ± 2	5.1 ± 2	0.736	5.3 ± 2	5.1 ± 2	0.502		5.5 ± 1.9	5.1 ± 2	0.472		5.5 ± 2	5 ± 2	0.199	
LSI (mean ± SD)	8.6 ± 1.1	7.8 ± 1.1	7.8 ± 1.2	0.098	8.1 ± 1.2	7.8 ± 1.2	0.221		8.6 ± 1.1	7.8 ± 1.2	0.031		8.2 ± 1.1	7.8 ± 1.2	0.038	
SLICC damage index (mean ± SD)	0.6 ± 0.9	1 ± 1.2	0.7 ± 1.2	0.616	0.8 ± 1.1	0.7 ± 1.2	0.715		0.6 ± 0.9	0.8 ± 1.2	0.590		0.8 ± 1.1	0.7 ± 1.2	0.582	

Comparisons were done using the Chi-square tests for the categorical data while the unpaired sample *t*-test and the one-way ANOVA test were used to compare the quantitative data between two and multiple groups, respectively. *n*: number. MC: mucocutaneous; MSK: musculoskeletal; NPS: neuropsychiatric; SDI: Lupus Severity of Disease Index; LSI: Lupus Severity Index; SLICC: Systemic Lupus International Collaborating Clinics; OR: odds ratio; CI: confidence interval.

## Data Availability

All available data are presented in this work.

## Additional Points

*Key Messages*. What is already known about this subject? Systemic lupus erythematosus (SLE) is a prototypic systemic autoimmune disease. Although several studies have tackled the disease pathogenesis, several risk factors, genetics and environmental, are still to be discovered. The autoimmune regulator (*AIRE*) is a master regulator of self-tolerance development. *AIRE* gene mutation and polymorphisms have been linked to several autoimmune disorders. To the best of our knowledge, very scarce data are available concerning this topic. What does this study add? The study revealed that *AIRE* polymorphisms, rs2075876 and rs760426, could add to SLE susceptibility, phenotypic expression, and burden in Egyptian patients. How might this impact on clinical practice? Understanding the detailed disease pathogenesis helps the implementation of preventive strategies in susceptible persons, and the development of targeted therapies in the affected individuals. *Highlights*. (i) *AIRE* polymorphism rs2075876 could add to SLE phenotypic expression in Egyptian patients. (ii) *AIRE* polymorphism rs760426 could add to SLE susceptibility in Egyptian patients. (iii) *AIRE* polymorphisms, rs rs2075876 and760426, could add to SLE burden in Egyptian patients.

## Supplementary Materials

Supplementary Materials Supplementary Table 1: the linkage disequilibrium analysis between the two *AIRE* polymorphisms.

## References

[1] D’Cruz D. P., Khamashta M. A., Hughes G. R. (2007). Systemic lupus erythematosus. *Lancet*.

[2] Housey M., DeGuire P., Lyon-Callo S. (2015). Incidence and prevalence of systemic lupus erythematosus among Arab and Chaldean Americans in Southeastern Michigan: the Michigan Lupus Epidemiology and Surveillance Program. *American Journal of Public Health*.

[3] Jugrin A. V., Sun Y., Cox F. (2014). The economic burden of systemic lupus erythematosus: a structured literature review. *Value in Health*.

[4] Marian V., Anolik J. (2012). Treatment targets in systemic lupus erythematosus: biology and clinical perspective. *Arthritis Research & Therapy*.

[5] Crow M. K., Firestein G. S., Budd R. C., Gabriel S. E., McInnes L. B., O’Dell J. R. (2017). Etiology and pathogenesis of systemic lupus erythematosus. *Kelley and Firestein’s Textbook of Rheumatology*.

[6] Kont V., Laan M., Kisand K., Merits A., Scott H. S., Peterson P. (2008). Modulation of Aire regulates the expression of tissue-restricted antigens. *Molecular Immunology*.

[7] Metzger T. C., Anderson M. S. (2011). Control of central and peripheral tolerance by AIRE. *Immunological Reviews*.

[8] Mathis D., Benoist C. (2009). AIRE. *Annual Review of Immunology*.

[9] Anderson M. S., Su M. A. (2011). AIRE and T cell development. *Current Opinion in Immunology*.

[10] Bruserud O., Oftedal B. E., Wolff A. B., Husebye E. S. (2016). _AIRE_ -mutations and autoimmune disease. *Current Opinion in Immunology*.

[11] Tazi-Ahnini R., McDonagh A. J. G., Wengraf D. A. (2008). The autoimmune regulator gene (AIRE) is strongly associated with vitiligo. *The British Journal of Dermatology*.

[12] Bonner S. M., Pietropaolo S. L., Fan Y. (2012). Sequence Variation in Promoter of Ica1 Gene, Which Encodes Protein Implicated in Type 1 Diabetes, Causes Transcription Factor Autoimmune Regulator (AIRE) to Increase Its Binding and Down-regulate Expression. *The Journal of Biological Chemistry*.

[13] Fierabracci A. (2011). The role of heterozygous mutations of the autoimmune regulator gene (AIRE) in non-APECED autoimmunity: a comment on recent findings. *Clinical Endocrinology*.

[14] Colobran R., Giménez-Barcons M., Marín-Sánchez A., Porta-Pardo E., Pujol-Borrell R. (2016). _AIRE_ genetic variants and predisposition to polygenic autoimmune disease: The case of Graves ' disease and a systematic literature review. *Human Immunology*.

[15] Lankisch T. O., Mourier O., Sokal E. M. (2009). AIRE gene analysis in children with autoimmune hepatitis type I or II. *Journal of Pediatric Gastroenterology and Nutrition*.

[16] Ströbel P., Chuang W.-Y., Chuvpilo S. (2008). Common cellular and diverse genetic basis of Thymoma-associated myasthenia Gravis. *Annals of the New York Academy of Sciences*.

[17] Wengraf D. A., McDonagh A. J. G., Lovewell T. R. J. (2008). Genetic analysis of autoimmune regulator haplotypes in alopecia areata. *Tissue Antigens*.

[18] Kuroda N., Mitani T., Takeda N. (2005). Development of autoimmunity against transcriptionally unrepressed target antigen in the thymus of AIRE-deficient mice. *Journal of Immunology*.

[19] Terao C., Yamada R., Ohmura K. (2011). The human AIRE gene at chromosome 21q22 is a genetic determinant for the predisposition to rheumatoid arthritis in Japanese population. *Human Molecular Genetics*.

[20] Shao S., Li X., Cen H., Yin Z. (2014). Association of AIRE polymorphisms with genetic susceptibility to rheumatoid arthritis in a Chinese population. *Inflammation*.

[21] Feng Z., Zhang S., Wena H., Liang Y. (2015). Association of rs2075876 polymorphism of AIRE gene with rheumatoid arthritis risk. *Human Immunology*.

[22] García-Lozano J.-R., Torres-Agrela B., Montes-Cano M.-A. (2013). Association of the AIRE gene with susceptibility to rheumatoid arthritis in a European population: a case control study. *Arthritis Research & Therapy*.

[23] Bérczi B., Gerencsér G., Farkas N. (2017). Association between AIRE gene polymorphism and rheumatoid arthritis: a systematic review and meta-analysis of case-control studies. *Sci Rep*.

[24] Xu Y., Jiang X., Chen J. (2017). A single nucleotide polymorphism of AIRE gene located in the 21q22.3 increases the risk of rheumatoid arthritis. *Oncotarget*.

[25] Lim J., Kim K. (2019). Genetic variants differentially associated with rheumatoid arthritis and systemic lupus erythematosus reveal the disease specific biology. *Scientific Reports*.

[26] Montufar-Robles I., Robles-Garnica J. C., Cadena-Sandoval D. (2019). The _AIRE_ Ser196Ser synonymous variant is a risk factor for systemic lupus erythematosus. *Cellular Immunology*.

[27] Alghamdi S. A., Kattan S. W., Toraih E. A., Alrowaili M. G., Fawzy M. S., Elshazli R. M. (2021). Association of _AIRE (rs2075876)_ , but not _CTLA4 (rs231775)_ polymorphisms with systemic lupus erythematosus. *Gene*.

[28] Petri M., Orbai A.-M., Alarcón G. S. (2012). Derivation and validation of the Systemic Lupus International Collaborating Clinics classification criteria for systemic lupus erythematosus. *Arthritis and Rheumatism*.

[29] Katz J. D., Senegal J., Rivest C., Goulet J., Rothfield N. (1993). A simple severity of disease index for systemic lupus erythematosus. *Lupus*.

[30] Bello G. A., Brown M. A., Kelly J. A., Thanou A., James J. A., Montgomery C. G. (2016). Development and validation of a simple lupus severity index using ACR criteria for classification of SLE. *Lupus Science & Medicine*.

[31] Lim J., Kim K. (1992). Systemic Lupus International Collaborative Clinics: development of a damage index in systemic lupus erythematosus. *The Journal of Rheumatology*.

[32] *Calculator of Hardy-Weinberg equilibrium*.

[33] *SNPStats: Your web tool for SNP analysis*.

[34] Ferrera F., Rizzi M., Sprecacenere B. (2007). _AIRE_ gene polymorphisms in systemic sclerosis associated with autoimmune thyroiditis. *Clinical Immunology*.

[35] Zhu M., Chin R. K., Christiansen P. A. (2006). NF-kappaB2 is required for the establishment of central tolerance through an AIRE-dependent pathway. *The Journal of Clinical Investigation*.

[36] Wong D., Teixeira A., Oikonomopoulos S. (2011). Extensive characterization of NF-*κ*B binding uncovers non-canonical motifs and advances the interpretation of genetic functional traits. *Genome Biology*.

[37] Stahl E. A., Consortium B. I. R. A. C., Raychaudhuri S. (2010). Genome-wide association study meta-analysis identifies seven new rheumatoid arthritis risk loci. *Nature Genetics*.

[38] Lovewell T. R., McDonagh A. J., Messenger A. G., Azzouz M., Tazi-Ahnini R. (2015). The AIRE -230Y polymorphism affects AIRE transcriptional activity: potential influence on AIRE function in the thymus. *PLoS One*.

[39] Rose A. B. (2008). Intron-mediated regulation of gene expression. *Current Topics in Microbiology and Immunology*.

[40] Yanagihara T., Sanematsu F., Sato T. (2015). Intronic regulation of _Aire_ expression by Jmjd6 for self-tolerance induction in the thymus. *Nature Communications*.

[41] MIYAKIS S., LOCKSHIN M. D., ATSUMI T. (2006). International consensus statement on an update of the classification criteria for definite antiphospholipid syndrome (APS). *Journal of Thrombosis and Haemostasis*.

[42] Lewiecki E. M., Gordon C. M., Baim S. (2008). International Society for Clinical Densitometry 2007 adult and pediatric official positions. *Bone*.

