# Supplementary material for: Autoimmune Regulator Gene Polymorphisms in Egyptian Systemic Lupus Erythematosus Patients: Preliminary Results
Source: Int J Rheumatol. 2021 Sep 28;2021:5546639. doi: 10.1155/2021/5546639 (PMC8492237; doi:10.1155/2021/5546639)
Supplement: Supplementary Materials — Supplementary Table 1: the linkage disequilibrium analysis between the two AIRE polymorphisms. [file 5546639.f1.docx]

**Table S1:** Haplotype analysis of *AIRE* rs2075876 and rs760426 in the study groups

| **Haplotype** | **rs2075876** | **rs760426** | **Healthy control** | **SLE patients** | **OR(95%CI)** | **P value** |
| --- | --- | --- | --- | --- | --- | --- |
| 1 | G | A | 375(56%) | 291(44%) | 1.00 | ----- |
| 2 | A | A | 247(54%) | 208(46%) | 0.87(0.5-1.5) | 0.59 |
| 3 | G | G | 220(54%) | 187(46%) | 0.7(0.4-1.2) | 0.15 |
| 4 | A | G | 92(46%) | 105(54%) | 11(1.7-72.6) | 0.014 |
| Global Haplotype association p-value: 0.00055 | | | | | | |
| Linkage Disequilibrium analysis between rs2075876 and rs760426 | | | | | | |
| *D*= 0.0105 *D’*=0.06666 *r^2^*= 0.0596 *P* value = 0.2232 | | | | | | |
